# Preconditioning With Natural Microbiota Strain *Ochrobactrum vermis* MYb71 Influences *Caenorhabditis elegans* Behavior

**DOI:** 10.3389/fcimb.2021.775634

**Published:** 2021-12-17

**Authors:** Carola Petersen, Barbara Pees, Christina Martínez Christophersen, Matthias Leippe

**Affiliations:** Department of Comparative Immunobiology, Zoological Institute, Christian-Albrechts University, Kiel, Germany

**Keywords:** microbiota, *C. elegans* natural isolate MY2079, choice behavior, avoidance response, *Ochrobactrum vermis* MYb71

## Abstract

In comparison with the standard monoxenic maintenance in the laboratory, rearing the nematode *Caenorhabditis elegans* on its natural microbiota improves its fitness and immunity against pathogens. Although *C. elegans* is known to exhibit choice behavior and pathogen avoidance behavior, little is known about whether *C. elegans* actively chooses its (beneficial) microbiota and whether the microbiota influences worm behavior. We examined eleven natural *C. elegans* isolates in a multiple-choice experiment for their choice behavior toward four natural microbiota bacteria and found that microbiota choice varied among *C. elegans* isolates. The natural *C. elegans* isolate MY2079 changed its choice behavior toward microbiota isolate *Ochrobactrum vermis* MYb71 in both multiple-choice and binary-choice experiments, in particular on proliferating bacteria: *O. vermis* MYb71 was chosen less than other microbiota bacteria or OP50, but only after preconditioning with MYb71. Examining escape behavior and worm fitness on MYb71, we ruled out pathogenicity of MYb71 and consequently learned pathogen avoidance behavior as the main driver of the behavioral change toward MYb71. The change in behavior of *C. elegans* MY2079 toward microbiota bacterium MYb71 demonstrates how the microbiota influences the worm’s choice. These results might give a baseline for future research on host–microbiota interaction in the *C. elegans* model.

## Introduction

The nematode *Caenorhabditis elegans* has been extensively used as a research model and is usually kept in monoxenic culture with the *Escherichia coli* strain OP50 as food. This is in stark contrast to *C. elegans* natural environment (e.g., rotting fruits, decomposing plant matter, and compost) where the nematode lives in close association with a huge variety of microbes (Félix and Braendle, 2010; Petersen et al., 2015; Dirksen et al., 2016). In the past years, research on the natural *C. elegans* microbiota revealed that the species-rich bacterial community of *C. elegans* is distinct from that of the corresponding natural environment and is dominated by Enterobacteriaceae and members of the genera *Pseudomonas*, *Stenotrophomonas*, *Ochrobactrum*, and *Sphingomonas* (Berg et al., 2016a; Dirksen et al., 2016; Samuel et al., 2016). Various microbiota genera are able to colonize and persist in the worm’s intestine and show beneficial effects on population growth and resistance against abiotic and biotic stress (Berg et al., 2016b; Dirksen et al., 2016; Samuel et al., 2016; Zimmermann et al., 2020; Pees et al., 2021). Protective effects of *Pseudomonas*, *Bacillus*, and *Enterobacter* strains against pathogen infection have been characterized (Montalvo-Katz et al., 2013; Berg et al., 2016b; Kissoyan et al., 2019). Furthermore, assessment of the inducible transcriptome response revealed influences of two *Ochrobactrum* strains on *C. elegans* dietary response, development, fertility, immunity, and energy metabolism indicating that further microbiota-mediated host functions are conceivable (Yang et al., 2019).

The influence of microbiota on vertebrate and invertebrate host behavior has been repeatedly demonstrated, for example, on olfactory behavior (Fischer et al., 2017; Qiao et al., 2019), microbial preference, and foraging decisions in *Drosophila melanogaster* (Wong et al., 2017) and social preference and depressive-like behavior in mice (Arentsen et al., 2015; Luo et al., 2018). In particular, natural *C. elegans* isolates show different behavioral responses depending on genotype, time of isolation from the wild, and sampling site (Volkers et al., 2013; Petersen et al., 2015). These behavioral responses are often regulated by the presence of food bacteria and can influence feeding, egg laying, and movement of the worm (Horvitz et al., 1982; Avery and Horvitz, 1990). Certain bacteria and small molecules can alter *C. elegans* chemotactic responses (Bargmann and Horvitz, 1991; Grewal and Wright, 1992; Shtonda and Avery, 2006). Differentiation between benign and harmful bacteria is particularly important under natural conditions in which worms face a multitude of microorganisms. Some natural *C. elegans* populations exhibit a preference for naturally coexisting bacterial species (Volkers et al., 2013) indicating that choice behavior may be involved in the identification and acquisition of symbionts, commensals, and microbiota (O’Donnell et al., 2020). However, information on the influence of the microbiota on *C. elegans* behavior is still limited.

The aim of this study was hence to identify and characterize the potential effects of the microbiota on the behavior of *C. elegans*. We focused on a trait of likely relevance under natural conditions, namely, choice behavior. We demonstrated that natural *C. elegans* strains differ in their choice behavior toward their microbiota bacteria. Particularly, preconditioning of natural *C. elegans* isolate MY2079 with proliferating *Ochrobactrum vermis* MYb71 resulted in a behavioral change upon a second exposure. Accordingly, colonizing MYb71 is able to alter *C. elegans* preference for the same bacterial strain. Our results contribute to the understanding of *C. elegans* behavior to its natural gut microbiota.

## Materials and Methods

### Nematode and Bacterial Strains

We used eleven natural *C. elegans* isolates (**Supplementary Table S1A**) isolated at two North German locations between 2002 and 2012 (Haber, 2005; Petersen et al., 2014) and the *C. elegans* laboratory strain N2. The natural isolates were chosen based on genotypic differences at the microsatellite level that translated into phenotypic variation (Petersen et al., 2015). All strains were maintained following standard procedures (Stiernagle, 2006).

Six bacterial strains were used as representatives for different genera of *C. elegans* native microbiota able to colonize the worm’s intestine (**Supplementary Table S1B**) (Dirksen et al., 2016; Dirksen et al., 2020; Zimmermann et al., 2020; Pees et al., 2021): the Gram-negative bacteria *Acinetobacter guillouiae* MYb10, *Pseudomonas fluorescens* MYb11, *Ochrobactrum anthropi* MYb49, *O. vermis* MYb71, and *Ochrobactrum pseudogrignonense* MYb237 and the Gram-positive bacterium *Rhodococcus erythropolis* MYb53 (Dirksen et al., 2016). Microbiota isolates were thawed freshly, cultured for 2 days on tryptic soy agar (TSA) at 25°C, and subsequently grown in tryptic soy broth (TSB) in a shaking incubator for 42 h at 28°C (MYb10, MYb49, MYb53, MYb71) or 16 h (MYb11). *E. coli* OP50 was cultured in TSB for 16 h at 37°C. For the experiments, all bacteria were adjusted to OD_600_10 in their supernatant if not stated otherwise.

### RNA Isolation

Bacterial lawns (OD_600_10) were grown for 24 h at 25°C on 9-cm nematode growth medium (NGM) plates. The lawns were scraped off, pooled from five plates, and pelleted at 4,000 *g* at 4°C for 15 min, and 100 µl of bacterial pellet was resuspended in 1 ml of RNAmagic (Bio-Budget Technology GmbH, Krefeld, Germany). Aliquots of 800 µl were frozen in liquid nitrogen, thawed at 45°C and frozen in three repeated cycles, and stored at −80°C until RNA isolation. Total RNA was isolated using MACHEREY-NAGEL (Düren, Germany) NucleoSpin Kit using the protocol for bacterial cells.

### Microbiota Choice Experiments

Choice behavior was examined using either multiple-choice experiments offering five bacterial strains simultaneously or binary-choice experiments with only two bacterial strains. *C. elegans* strains were either unconditioned or preconditioned with a specific microbiota bacterium. Worms touching a bacterial spot were scored, and all other worms were not taken into account. The position of the bacteria across assay plates was randomized, and all experiments were performed without current knowledge of worm and bacterial strain identity to avoid any observer bias.

### Multiple-Choice Experiment

For the multiple-choice experiments, four microbiota bacteria and OP50 were adjusted to OD_600_10 in phosphate-buffered saline (PBS), and 25 µl of each bacterium and a PBS control were pipetted equidistantly in a circle onto 9-cm peptone-free NGM (PFM) plates or NGM plates and left to dry for 2 h. Approximately 100 synchronized *C. elegans* in the fourth larval stage (L4) were pipetted to the center of the plate. The number of worms residing on each bacterium was determined after 2 or 24 h. We calculated the mean proportion of worms per bacterium = number of worms on a bacterium/total number of worms on all bacteria per plate.

### Preconditioning of *Caenorhabditis elegans*

*C. elegans* was grown on OP50 until the L4 stage, washed three times in sterile M9 buffer, and pipetted to preconditioning plates freshly inoculated with one bacterium in OD_600_10. Preconditioning was performed with live and heat-killed (80°C for 40 min) MYb71 and OP50, with OP50 lawns spiked with 21 ng of total RNA of MYb71 or OP50, or with the odor of viable MYb71 or OP50. For the latter, L4 worms were placed on OP50 lawns with either 500 µl MYb71 or OP50 on a thin NGM layer in the lid of the plate. Lining the edge of the plate with 1% palmitic acid in ethanol kept the worms on the OP50 lawn. After 24 h, the preconditioned adults were washed repeatedly in M9 buffer and subsequently applied to binary-choice assay plates.

### Binary-Choice Assay

Approximately 70 naïve L4 worms or preconditioned adults were pipetted centrally between two opposing 30-µl bacterial spots on 6-cm NGM plates. The number of worms residing on each bacterium was determined after 2 and/or 24 h. We calculated a choice index = (number of worms on the bacterium opposing the microbiota bacterium − number of worms on the microbiota bacterium)/(total number of worms on both bacteria). To test for bacterial signals produced upon contact with worms, the initially placed worms were removed after 24 h, and naïve adult worms (from OP50) were pipetted centrally onto the same plates. The choice index for the newly introduced worms was determined after 2 h (26 h total time).

### Bacterial Lawn Leaving Experiment

Synchronized *C. elegans* MY2079 L4 worms were preconditioned on MYb71 or OP50 for 24 h. Adults were washed repeatedly in M9 buffer, and approximately 50 adults were pipetted onto NGM plates inoculated with 50 µl of either MYb71 or OP50. The number of worms on and outside the bacterial lawn was scored after 2 h. We calculated the proportion of escaped worms = number of worms outside the lawn/total number of worms on the plate.

### Brood Size Assay

NGM and PFM plates were inoculated daily with 50 µl MYb71 or OP50 at OD_600_10 adjusted in PBS and stored at 20°C for 24 h. Initially, one L4 worm was picked onto the plate and transferred daily to a new plate. We scored the hatched offspring of each worm until the worm stopped laying eggs. Data of worms that died or escaped from the plate before the end of the egg-laying period were excluded.

### Statistics

Statistical calculations were performed with R studio software (version 1.3.1093) and can be found in **Supplementary Table S2**. Graphs were produced with R Studio (version 1.3.1093) and edited with Inkscape (version 1.0.1).

## Results

### Microbiota Bacteria Influence the Choice Behavior of Natural *Caenorhabditis elegans* Isolate MY2079

**Figure 1:**
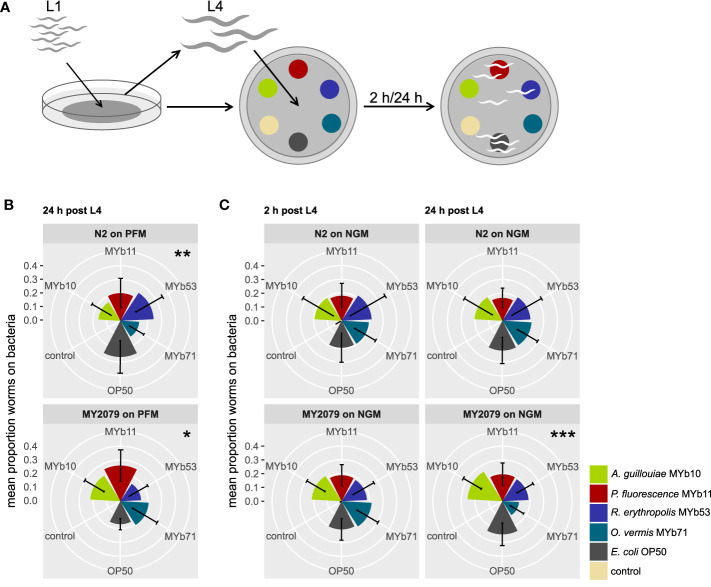
Natural *Caenorhabditis elegans* isolate MY2079 alters its choice behavior on proliferating microbiota after 24h. **(A)** Synchronized *C*. *elegans* were grown on OP50 until L4 stage and then used in multiple-choice experiments. The choice behavior of *C. elegans* N2 and MY2079 was evaluated toward a subset of **(B)** non-proliferating microbiota on peptone-free medium (PFM) after 24 h and **(C)** proliferating microbiota on nematode growth medium (NGM) after 2 and 24 h post L4. Shown are means ± SD of n = 15 (with ~100 worms per n), Kruskal–Wallis (Kruskal and Wallis, 1952) with false discovery rate correction for multiple testing (Benjamini and Hochberg, 1995); asterisks indicate difference within the given treatment. Significance is designated to the following scale: ***p < 0.001, **p < 0.01, *p < 0.05.

N2 can be considered as a laboratory animal, domesticated to a monoxenic environment with only *E. coli* OP50 as a food source. Natural *C. elegans* isolates can differ in their behavioral response to bacterial cues (Petersen et al., 2015; Pradhan et al., 2019). Since *C. elegans* microbiota was originally extracted from natural *C. elegans* isolates, we selected eleven genotypically divergent natural *C. elegans* isolates (Petersen et al., 2015) and the laboratory strain N2 to screen their choice behavior toward four microbiota isolates from different genera and OP50 as control (**Figure 1A**).

We defined choice behavior as present if worms preferred at least one of the offered bacteria over the others. In an initial screen, we identified general significant choice behavior after 24 h in one natural *C. elegans* isolate, MY2079 (*p* = 0.022; **Supplementary Figure S1**), and a trend in eight natural *C. elegans* isolates (*p* < 0.063). We confirmed the results of the screen for MY2079 with an additional multiple-choice experiment using a higher replication (n = 15), in which not only MY2079 (*p* = 0.012) but this time also N2 (*p* = 0.008) showed choice behavior after 24 h. Yet the choice behavior between MY2079 and N2 differed significantly (*p* < 0.001; chi-squared test) (**Figure 1B**).

In its ephemeral natural habitats, *C. elegans* lives a boom-and-bust life cycle (Frézal and Félix, 2015), because as the availability of plant material varies, so do the growth conditions and the availability of food organisms. Hence, we wondered whether the availability of nutrients for bacterial growth influences the choice behavior toward microbiota. We examined the effect of bacterial proliferation on the choice behavior of *C. elegans* N2 and the natural isolate MY2079 in a multiple-choice experiment on NGM, which supports bacterial proliferation. *C. elegans* MY2079 showed choice behavior after 24 h (*p* < 0.001) but not after 2 h, whereas *C. elegans* N2 showed generally no choice behavior on NGM (**Figure 1C**). Further, the choice behavior between *C. elegans* MY2079 and N2 on NGM differed significantly after 2 h (*p* < 0.001; chi-squared test) and 24 h (*p* < 0.001; chi-squared test) (**Figure 1C**).

These results indicate that the choice behavior of N2 and MY2079 differs and that *C. elegans* MY2079 shows choice behavior particularly in the presence of proliferating microbiota.

### Natural *Caenorhabditis elegans* Isolate MY2079 Avoids *Ochrobactrum vermis* MYb71 Over Time

**Figure 2:**
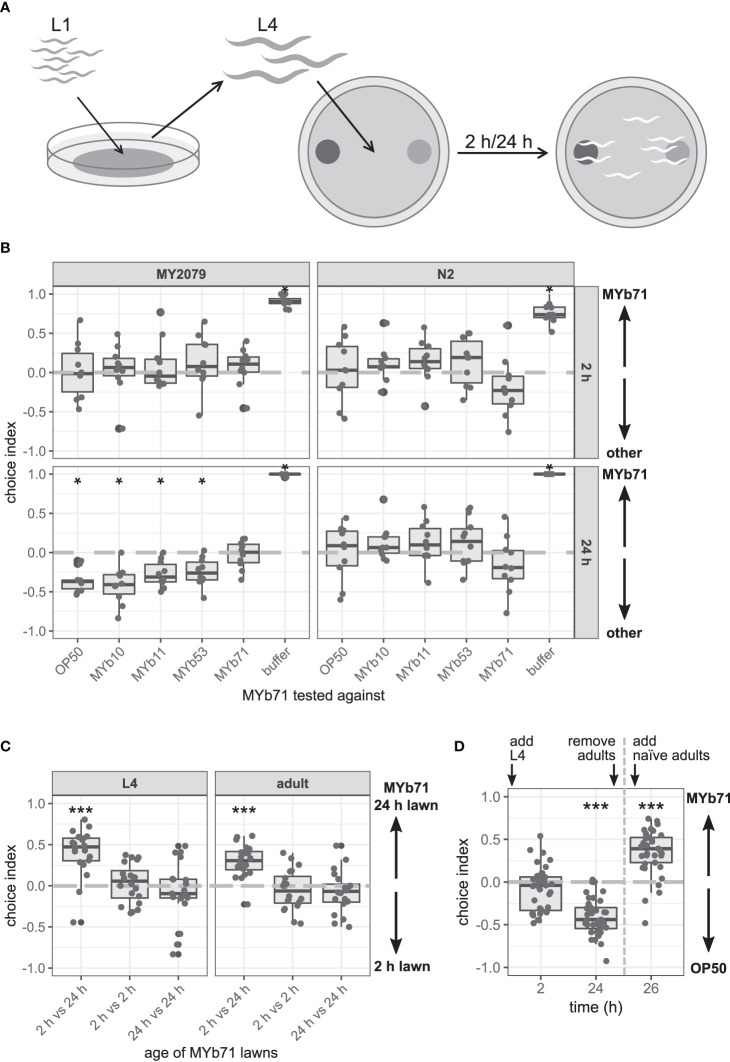
The late microbiota avoidance response is specific to MYb71, independent of MYb71 lawn age, and is not shown in naïve MY2079. **(A)** Synchronized *Caenorhabditis elegans* were grown on OP50 until L4 stage and then used in binary-choice experiments using MYb71 (dark gray) vs. a test bacterium (light gray). The choice index was determined at 2 and 24 h post L4. **(B)** The choice behavior of *C. elegans* N2 and MY2079 toward MYb71 was tested against individual microbiota isolates or OP50 (n = 10, with ~70 worms per n). **(C)** Choice behavior of MY2079 L4 larvae and adults toward a “new” (2 h) and “old” (24 h) lawn (n = 20, with ~70 worms per n). **(D)** MYb71 and OP50 on choice plates were exposed to MY2079 worms, and the choice behavior of MY2079 was evaluated after 2 and 24 h. After removal of the initially placed worms, the choice behavior of naïve adults was evaluated after 2 h on the same plates (n = 34-40, with ~70 worms per n). A negative choice index indicates a choice of **(B)** OP50, MYb10, MYb11, MYb53, or a buffer control; or **(C)** the 2 h lawn; or **(D)** OP50. A positive choice index indicates a choice of **(B, D)** MYb71 or **(C)** the 24 h MYb71 lawn. A choice index of 0 indicates the equal choice of bacteria on both sides. Wilcoxon signed-rank test (Wilcoxon, 1945) with false discovery rate (FDR) correction for multiple testing was applied for comparing the choice indices to 0. Data are presented in boxplots; each gray dot represents one replicate. Significance is designated to the following scale: ***p < 0.001, *p < 0.05.

We noticed in the multiple-choice experiment on proliferating bacteria that MY2079 changed its choice behavior toward MYb71 and OP50 over time and eventually avoided MYb71 in favor of OP50 (**Figure 1C**). Hence, we wondered whether the avoidance behavior toward MYb71 is due to repulsion from MYb71 or increased attraction toward one of the other bacteria. We performed a binary-choice experiment with MYb71 opposing one test bacterial strain at a time (**Figure 2A**). *C. elegans* N2 did not change its choice behavior over time and chose MYb71 and the opposing test bacteria equally. Similarly, *C. elegans* MY2079 chose MYb10, MYb11, MYb53, and OP50 equally to MYb71 at 2 h (**Figure 2B**). However, after 24 h, MY2079 preferred all other bacteria over MYb71 (*p* = 0.028). These results suggest that the change in choice behavior toward MYb71 over time is specific for *C. elegans* MY2079 and is caused by avoidance of proliferating MYb71.

### Bacterial Cell Density and Worm Developmental Stage are not Responsible for Late Microbiota Avoidance Response

What might be responsible for the change in the choice behavior of MY2079 toward MYb71 over time – a phenomenon that we as of now call “late microbiota avoidance response”? In the course of the choice experiment, two main factors changed: 1) bacterial proliferation led to an increase in cell density and therefore higher concentration of bacterial products after 24 compared with 2 h. 2) At the same time, the worms developed from the L4 stage to the adult stage. We therefore compared the attraction of *C. elegans* MY2079 toward MYb71 in OD_600_10 and OD_600_30 (directly adjusted from a liquid culture) and toward MYb71 lawns grown on a plate for 2 or 24 h. The latter was done for L4 larvae and adults to examine developmental influences. Both L4 larvae (*p* < 0.001) and adults (*p* < 0.001) chose the 24 h MYb71 lawn over the 2 h lawn (**Figure 2C**). Similarly, L4 larvae of N2 (*p* = 0.003) and MY2079 (*p* = 0.014) chose MYb71 at OD_600_30 over OD_600_10 (**Supplementary Figure S2**).

These findings indicate that *C. elegans* prefers a higher MYb71 cell density in direct comparison with a lower MYb71 cell density independent of the developmental stage. However, in the presence of an alternative bacterial lawn, the MYb71 lawn after 24 h is less attractive (**Figure 2B**). Therefore, an increased cell density in the course of the experiment did not explain the late microbiota avoidance response toward MYb71 in the previous experiments.

### The Late Microbiota Avoidance Response Is Independent of Worm-Exposed *Ochrobactrum vermis* MYb71

Bacterial communities are able to react to their environment, including being grazed on by bacterivores. *Polynucleobacter asymbioticus*, for example, changes its transcriptional profile upon grazing by chrysophytes concerning transcriptional, translational, and stress responses (Beisser et al., 2019). These transcriptional differences in bacteria and resulting changes in the availability of bacterial products might be perceived by *C. elegans* and consequently influence its behavior. To test the potential production of worm-repelling compounds after worm exposure, we exposed MYb71 to *C. elegans* in a binary-choice experiment, removed the worms after 24 h, and exposed naïve worms to the same bacteria. As seen before, the initially placed *C. elegans* showed the late microbiota avoidance response toward MYb71 (*p* < 0.001; **Figure 2D**). Interestingly, naïve worms did not avoid the 24-h-old, worm-exposed MYb71 but preferred this lawn over OP50 instead (*p* < 0.001).

These findings suggest that the production of a repellent bacterial product triggered by exposure to worms is rather unlikely.

### The Late Microbiota Avoidance Response Can Be Induced by Preconditioning Worms With MYb71 and Is Specific to MYb71

**Figure 3:**
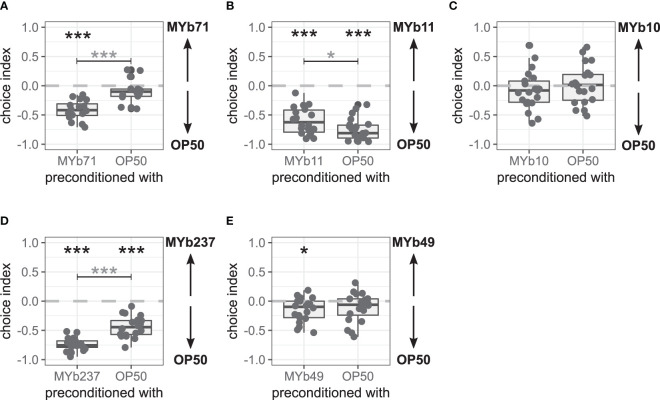
Late microbiota avoidance response of *Caenorhabditis elegans* after bacterial preconditioning is species-specific. Choice behavior of *C. elegans* MY2079 2 h after preconditioning with **(A)** OP50 or microbiota *Ochrobactrum vermis* MYb71 (n = 16, with ~70 worms per n), **(B)** *Pseudomonas lurida* MYb11 (n = 22, with ~70 worms per n), **(C)** *Acinetobacter guillouiae* MYb10 (n = 22, with ~70 worms per n), **(D)** *Ochrobactrum pseudogrignonense* MYb237 (n = 20, with ~70 worms per n), or **(E)** *Ochrobactrum anthropi* MYb49 (n = 20, with ~70 worms per n). Additional runs are shown in **Supplementary Figure S3**. Wilcoxon signed-rank test with false discovery rate (FDR) correction for multiple testing was applied for comparing the choice indices to 0. Data are presented in boxplots; each gray dot represents one replicate. A negative choice index indicates a choice of OP50, a positive choice index indicates a choice of the microbiota, and a choice index of 0 indicates the equal choice of both bacteria. Asterisks indicate a difference to a choice index of 0 (black) or a difference between preconditioning treatments (gray). Significance is designated to the following scale: ***p < 0.001, *p < 0.05.

As we did not observe a late microbiota avoidance response in naïve worms but in worms that have likely fed on MYb71, we hypothesized that worms had to be exposed to MYb71 to show the late microbiota avoidance response. Therefore, we preconditioned worms with MYb71 or OP50, i.e., exposed L4 worms for 24 h to either MYb71 or OP50, and subsequently performed a binary-choice experiment scoring behavior only after 2 h. Interestingly, we found the late microbiota avoidance response in preconditioned worms as in previous assays after 24 h, whereas worms preconditioned with OP50 did not show the late microbiota avoidance response (*p* < 0.001; **Figure 3A** and **Supplementary Figures S3A, B**). This indicates that preconditioning with MYb71 is enough to induce the late microbiota avoidance response.

We further wondered whether the choice behavior changed due to general intestinal colonization with microbiota and examined the choice behavior after preconditioning with two other representatives of colonizing microbiota, *P. lurida* MYb11 and *A. guillouiae* MYb10. *C. elegans* MY2079 chose OP50 over MYb11 independent of the preconditioning (*p* < 0.001; **Figure 3B** and **Supplementary Figures S3C, D**). However, worms preconditioned with MYb11 chose MYb11 slightly more compared with worms preconditioned with OP50 (*p* = 0.014). Preconditioning with MYb10 had no effect on choice behavior, and MYb10 and OP50 were always chosen equally (**Figure 3C** and **Supplementary Figures S3E, F**).

We used two additional *Ochrobactrum* microbiota isolates, *O. pseudogrignonense* MYb237 and *O. anthropi* MYb49, to test for a genus-specific preconditioning effect on choice behavior. *C. elegans* MY2079 chose OP50 over MYb237 independent of the preconditioning (*p* < 0.001), but OP50 was even more preferred in worms preconditioned with MYb237 (*p* < 0.001; **Figure 3D** and **Supplementary Figure S3G**). Preconditioning with MYb49 led to the choice of OP50 over MYb49 (*p* = 0.014), whereas worms preconditioned with OP50 chose OP50 and MYb49 equally in two out of three runs (**Figure 3E** and **Supplementary Figures S3H, I**). Overall, preconditioning with MYb49 compared with preconditioning with OP50 had no effect on the choice behavior.

These results support that preconditioning with colonizing microbiota can influence *C. elegans* choice behavior. However, neither colonization with microbiota nor preconditioning with bacteria of the genus *Ochrobactrum* resulted in a late avoidance response of the microbiota, in which avoidance occurred only after preconditioning with the test bacterium. Hence, the late microbiota avoidance response appears to be specific for *O. vermis* MYb71.

### The Late Microbiota Avoidance Response Is Independent of Bacterial RNA but Dependent on MYb71 Colonization of the Worm

Pathogenic bacteria, bacteria with insufficient nutritional value, or bacteria that negatively affect fitness in any other way can be avoided by *C. elegans* in order to reduce their uptake (Schulenburg and Müller, 2004; Meisel and Kim, 2014). Since we observed avoidance of MYb71, we evaluated whether the late microbiota avoidance response is linked to potential pathogenicity of MYb71. We found neither any lawn leaving behavior from MYb71 (**Supplementary Figure S4A**) nor a detrimental effect of MYb71 on the worm’s fitness (**Supplementary Figure S4B**). These observations suggest that MYb71 does not have a pathogenic effect on *C. elegans*.

Even without obvious pathogenicity of MYb71 to *C. elegans*, the question still remains whether *C. elegans* displays learned avoidance behavior mediated by bacterial products or whether gut colonization is necessary to elicit the worm’s late microbiota avoidance response.

**Figure 4:**
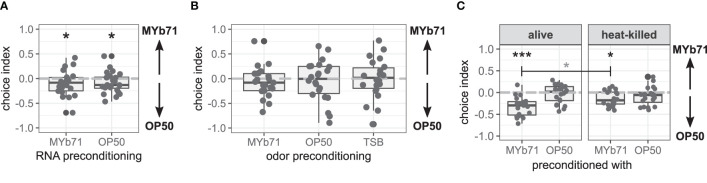
Late microbiota avoidance response is independent of total RNA of MYb71 or MYb71 odor but is more pronounced with viable MYb71. **(A)** Choice behavior after preconditioning with RNA of MYb71 or OP50 applied to viable OP50 (n = 24, with ~70 worms per n); or **(B)** after preconditioning with the odor of MYb71, OP50, or TSB (n = 22, with ~70 worms per n); or **(C)** after preconditioning with alive or heat-killed MYb71 or OP50 (n = 20, with ~70 worms per n). A negative choice index indicates the choice of OP50, a positive choice index indicates the choice of MYb71, and a choice index of 0 indicates the equal choice of both bacteria. Wilcoxon signed-rank test, false discovery rate (FDR)-corrected. Data are presented in boxplots; each gray dot represents one replicate. Asterisks indicate a difference to a choice index of 0 (black) or difference between treatments (gray). Significance is designated to the following scale: ***p < 0.001, *p < 0.05.

Recently, the learned pathogen avoidance behavior of *C. elegans* against *Pseudomonas aeruginosa* PA14 has been demonstrated to depend on a single non-coding RNA derived from PA14 (Kaletsky et al., 2020; Moore et al., 2021). Hence, we tested the influence of preconditioning with total RNA of MYb71 or OP50 on choice behavior and found that worms from both treatments chose OP50 over MYb71 in a binary choice assay (*p* = 0.044, **Figure 4A**).

The learned pathogen avoidance response against PA14 is further mediated by bacteria-derived odors (Zhang et al., 2005). Here, worms preconditioned with MYb71 odor chose MYb71 and OP50 equally independent of the bacteria used for odor preconditioning (**Figure 4B**).

To answer the question of whether *C. elegans* needs to be colonized by MYb71, we tested the late microbiota avoidance response of worms preconditioned with either heat-killed bacteria or viable bacteria. Both worms preconditioned with viable (*p* < 0.001) and heat-killed MYb71 (*p* = 0.011) chose OP50 over MYb71 (**Figure 4C**). However, worms preconditioned with viable MYb71 resided more often on OP50 lawns than worms preconditioned with heat-killed MYb71 (*p* = 0.022). Worms from OP50 chose MYb71 and OP50 equally, independent of the preconditioning.

These results indicate that the late microbiota avoidance response is not caused by total bacterial RNA nor bacteria-derived odors but primarily by the worm’s colonization with viable MYb71.

## Discussion

In the present study, we examined the choice behavior of the bacterivore nematode *C. elegans* toward its natural microbiota. We found that the natural *C. elegans* isolate MY2079 avoided specifically the microbiota isolate *O. vermis* MYb71 during a second exposure.

We observed the microbiota avoidance response of *C. elegans* MY2079 toward *O. vermis* MYb71 only either over time or during a second encounter. This behavior is reminiscent of the learned avoidance behavior in *C. elegans* against the pathogenic *P. aeruginosa* PA14 (Zhang et al., 2005). *C. elegans* trained for at least 4 h on PA14 lawns avoids PA14 during a second encounter more than untrained animals. On the host side, a complex interaction of TGFβ signaling (Zhang and Zhang, 2012; Meisel et al., 2014; Moore et al., 2019; Singh and Aballay, 2019), serotonin signaling (Zhang et al., 2005), and RNAi and piRNA signaling (Kaletsky et al., 2020) mediates the learned avoidance behavior. On the microbe side, bacterial secondary metabolites (Meisel et al., 2014) and non-coding RNAs (Kaletsky et al., 2020) trigger the learned behavioral response – in the case of non-coding RNA even across multiple worm generations (Kaletsky et al., 2020). Interestingly, so far only pathogenic bacteria such as PA14, *Serratia marcescens* ATCC 13880 (Zhang et al., 2005; Zhang and Zhang, 2012), or *Enterococcus faecalis* (Filipowicz et al., 2021) were known to elicit the learned avoidance behavior. *Ochrobactrum*, however, has been repeatedly isolated from wild *C. elegans* and has positive or neutral effects on *C. elegans* life-history traits such as population growth (Zimmermann et al., 2020), animal growth rates, and body size (Dirksen et al., 2020; Zhang et al., 2021). *Ochrobactrum* is therefore considered to be part of the natural, benign *C. elegans* microbiome (Berg et al., 2016a; Dirksen et al., 2016; Dirksen et al., 2020). The absence of lawn leaving behavior and lack of negative effects on brood size of *C. elegans* MY2079 presented in our study together with the mentioned published data support the benign nature of MYb71. Moreover, we excluded the small RNA-mediated mechanism of learned pathogen avoidance behavior (Kaletsky et al., 2020). Our finding is, hence, the first demonstration of an altered avoidance response after the first encounter as a reaction to a benign microbiota member. To explore to what extent such an avoidance response might benefit *C. elegans* MY2079 would be an interesting future direction.

Gut colonization with certain bacteria has been shown to alter *C. elegans* behavior in the context of microbiome bacteria: gut-colonizing *Providencia* bacteria manipulate host behavior by the production of the neuromodulator tyramine. Consequently, these bacteria are preferentially selected in food choice assays if *C. elegans* is colonized by *Providencia* (O’Donnell et al., 2020). Comparing preconditioning with heat-killed and viable MYb71, we showed that the avoidance was reduced when MYb71 was metabolically inactive. Therefore, bacteria-derived products could play a role in our observed late microbiota avoidance response. Further, *O. vermis* MYb71 colonizes and persists very efficiently in *C. elegans* (Dirksen et al., 2016; Zimmermann et al., 2020; Pees et al., 2021), providing the basis for host–microbe communication. Colonization by viable MYb71 is important for the late microbiota avoidance response because the worms showed no late avoidance response after exposure to MYb71 odor and reduced avoidance after exposure to heat-killed MYb71. As host response, MY2079 may synthesize substances responsible for the late microbiota avoidance response only when its gut is colonized by MY71. Moreover, the colonization with MYb71 might lead to a gut distention provoking the late microbiota avoidance response. The learned pathogen avoidance response against *E. faecalis* is caused by its accumulation in the anterior worm gut mediated by two transient receptor potential melastatin channels – even with an attenuated pathogen (Filipowicz et al., 2021).

Bacterial colonization of the *C. elegans* gut alone is certainly not the only trigger for the late microbiota avoidance response, as none of the other tested colonizing microbiota members phenocopied the response identified toward MYb71. The late microbiota avoidance response exhibits a very specific host–microbe interaction whose exchanged information and communicational signals remain to be discovered.

On the host side, the late microbiota avoidance response was specific for one natural *C. elegans* isolate, MY2079. In our initial multiple-choice screen, neither laboratory-adapted wild-type N2 nor the other 10 tested natural isolates showed significant choice behavior, i.e., did not prefer one of the offered bacterial spots over the others. However, the results of the multiple-choice screen are based on a relatively small number of replicates and many multiple comparisons. For the follow-up experiments, the number of replicates was greatly increased, and the number of comparisons was reduced, which then even led to significant choice behavior in N2, yet only on non-proliferating bacteria. The differences in choice behavior among the wild isolates exemplify the phenotypic diversity in *C. elegans* wild isolates as previously described (Petersen et al., 2015). The more profound differences in the behavioral response between N2 and MY2079 toward MYb71, however, can be attributed to the adaptation of N2 to laboratory conditions. Decades of maintenance on solely *E. coli* OP50 made the ability to choose specific microbiota bacteria increasingly redundant. A mutation in the G protein-coupled neuropeptide receptor gene *npr-1*, which leads to a solitary, non-bordering phenotype in N2 compared with a clumping and bordering phenotype in natural *C. elegans* isolates (de Bono and Bargmann, 1998; Reddy et al., 2009; Weber et al., 2010; Andersen et al., 2014), is only one example of the adaption of N2 to the laboratory.

In conclusion, we found a novel avoidance response of the natural *C. elegans* isolate MY2079 specifically toward the microbiota isolate *O. vermis* MYb71. The exact factors of this inter-kingdom communication remain to be determined. Our findings, however, might initiate further research on how the behavior of *C. elegans* is influenced by its natural gut microbiota.

## Data Availability Statement

The raw data supporting the conclusions of this article will be made available by the authors, without undue reservation.

## Author Contributions

CP: conceptualization, data curation, formal analysis, investigation, methodology, visualization, supervision of CMC, writing – original draft preparation, and writing – review and editing. BP: conceptualization, data curation, formal analysis, investigation, methodology, writing – original draft preparation, and writing – review and editing. CMC: investigation and methodology. ML: resources and funding acquisition. All authors contributed to the article and approved the submitted version.

## Funding

The work was funded by the German Science Foundation within the Collaborative Research Center CRC 1182 on Origin and Function of Metaorganisms (project A1.3 to ML and project A1.1 funding for CP). The funders had no role in study design, data collection and analysis, decision to publish, or preparation of the manuscript.

## Conflict of Interest

The authors declare that the research was conducted in the absence of any commercial or financial relationships that could be construed as a potential conflict of interest.

## Publisher’s Note

All claims expressed in this article are solely those of the authors and do not necessarily represent those of their affiliated organizations, or those of the publisher, the editors and the reviewers. Any product that may be evaluated in this article, or claim that may be made by its manufacturer, is not guaranteed or endorsed by the publisher.
