# Supplementary material for: Preconditioning With Natural Microbiota Strain Ochrobactrum vermis MYb71 Influences Caenorhabditis elegans Behavior
Source: Front Cell Infect Microbiol. 2021 Dec 17;11:775634. doi: 10.3389/fcimb.2021.775634 (PMC8718863; doi:10.3389/fcimb.2021.775634)
Supplement: Supplementary file 1 [file DataSheet_1.pdf]

Supplementary Figures

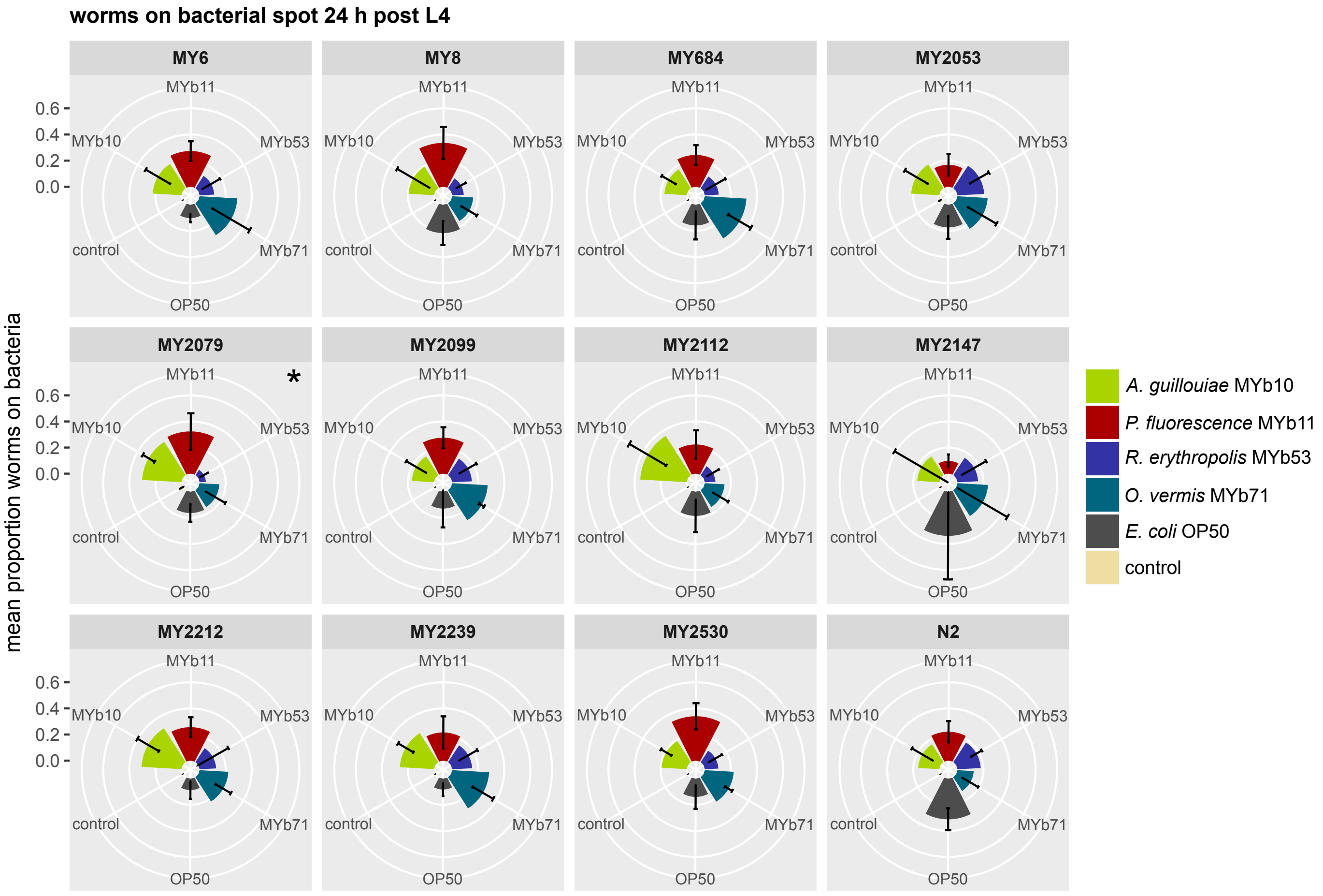

Supplementary Figure S1: *C. elegans* natural isolate MY2079 shows choice behavior in a multiple-choice experiment. The choice behavior of twelve *C. elegans* strains was tested in a multiple-choice setup using four microbiota bacteria, *E. coli* OP50, and PBS (control). The proportion of worms on each bacterium was evaluated after 24 h. Shown are means +/- standard deviation of n = 5 (with ~100 worms per n), Kruskal-Wallis with false discovery rate correction for multiple testing, asterisk indicates difference within the given strain. Significance is designated to the following scale: * $p < 0.05$.

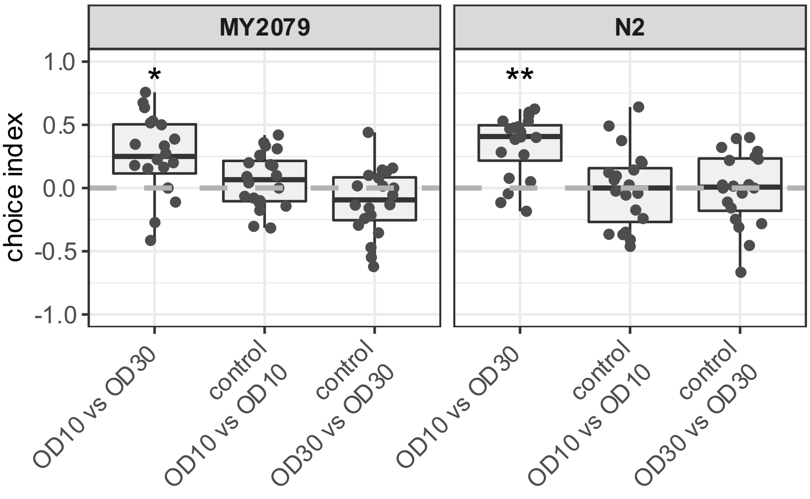

Supplementary Figure S2: *C. elegans* chooses the bacterial spot with higher density. The choice behavior of *C. elegans* N2 and MY2079 in the presence of two optical densities of MYb71 was evaluated after 2 h. A negative choice index indicates choice of MYb71 in OD_{600}10, a positive choice index indicates choice of MYb71 in OD_{600}30, a choice index of 0 indicates equal choice of both sides. Lawns with equal OD_{600} were offered as controls. Each grey dot represents one replicate (n = 20, with ~70 worms per n), Wilcoxon signed rank test (FDR-corrected). Asterisks indicate difference to choice index of 0. Significance is designated to the following scale: ** $p < 0.01$, * $p < 0.05$.

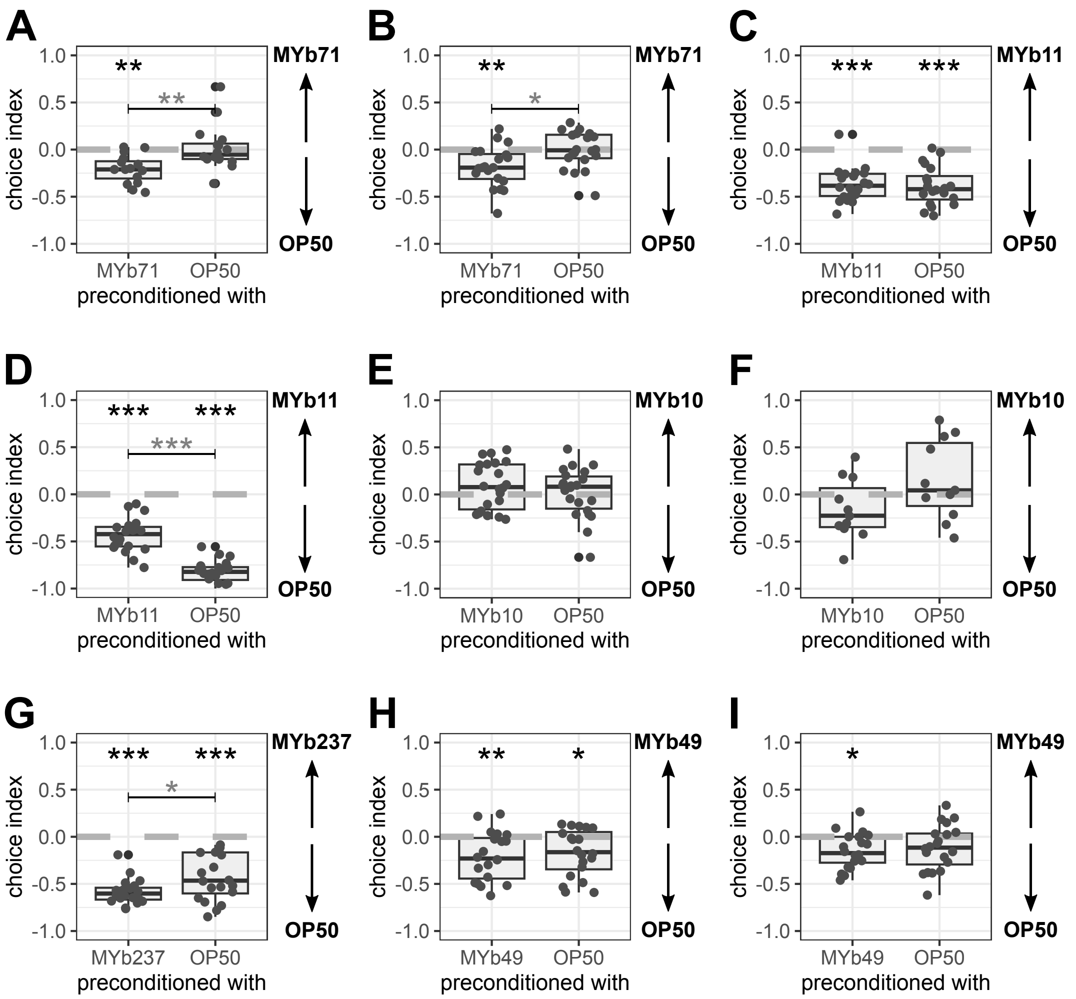

Supplementary Figure S3: Late microbiota avoidance response of *C. elegans* after bacterial preconditioning is species-specific. Additional runs to test choice behavior of *C. elegans* MY2079 2 h after preconditioning with OP50 or microbiota **(A, B)** *O. vermis* MYb71 (n = 16-20), **(C, D)** *P. lurida* MYb11 (n = 20), **(E, F)** *A. guillouiae* MYb10 (n = 11-22), **(G)** *O. pseudogrignonense* MYb237 (n = 20), or **(H, I)** *O. anthropi* MYb49 (n = 20). Approximately 70 worms were used per n. Wilcoxon signed rank test with FDR correction for multiple testing was applied for comparing the choice indices to 0 (Supplementary Table S2). Data are presented in boxplots; each grey dot represents one replicate. A negative choice index indicates choice of OP50, a positive choice index indicates choice of the microbiota, a choice index of 0 indicates equal choice of both bacteria. Asterisks indicate difference to a choice index of 0 (black) or difference between preconditioning treatments (grey). Significance is designated to the following scale: *** $p < 0.001$, * $p < 0.01$, * $p < 0.05$.

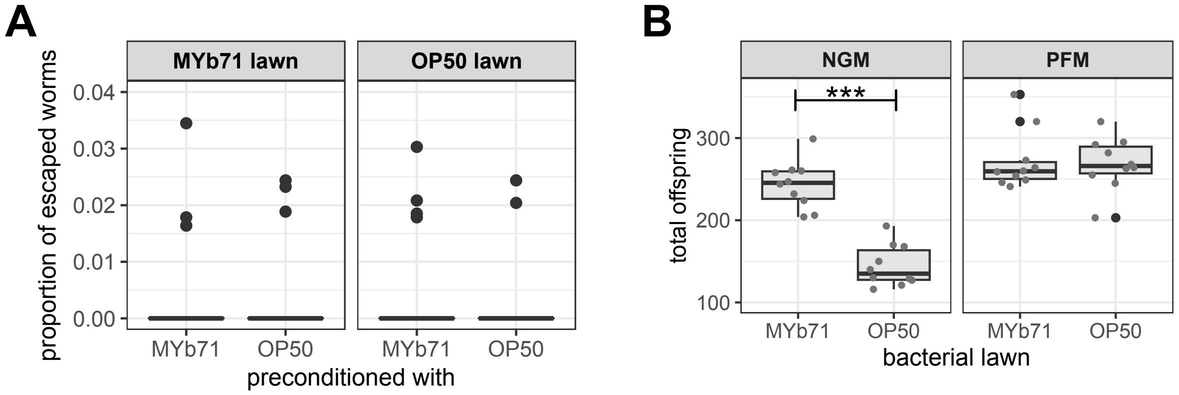

Supplementary Figure S4: *O. vermis* MYb71 does not have detrimental effects on *C. elegans*. (A) Comparison of the proportion of *C. elegans* MY2079 worms that left the *O. vermis* MYb71 or OP50 lawn after preconditioning with MYb71 or OP50 (n = 22, with ~50 worms per n). **(B)** Total brood size of *C. elegans* MY2079 was measured as sum of all hatched offspring produced by one worm either on peptone-free medium (PFM) or nematode growth medium (NGM) (n = 10). The total brood size corresponded to the sum of all hatched worms. Wilcoxon signed rank test. Data are presented in boxplots; each grey dot represents one replicate. Asterisks indicate significant difference between treatments. Significance is designated to the following scale: *** $p < 0.001$.
